# Semaglutide-induced lean mass loss: clinical concern or physiological adaptation

**DOI:** 10.1097/MS9.0000000000005145

**Published:** 2026-05-11

**Authors:** Muhammad Qaseem, Naveed Ahmad, Ehtisham Haider, Abdul Haseeb Hasan

**Affiliations:** a Department of Medicine, Khyber Medical College, Peshawar, Pakistan; b Department of Medicine, Punjab Medical College, Faisalabad, Pakistan; c Department of Research, Oli Health Magazine Organization, Research and Education, Kigali, Rwanda; d Department of Internal Medicine, Sentara Northern Virginia Medical Center, VA, USA; e Department of Medicine, King Edward Medical University, Lahore, Pakistan

*Dear Editor*,

Obesity is a chronic, multifactorial condition characterized by excessive fat buildup, which is detrimental to health, typically defined as a BMI of 30 kg/m^2^ or greater, and extreme obesity as 40 kg/m^2^ or greater. It occurs through a complicated process involving disturbed energy balance, genetic factors, hormonal influences, chronic low-grade inflammation, and changes in the gut–brain axis. Clinically, it is characterized by the accumulation of abdominal fat, insulin resistance, dyslipidemia, hypertension, sleep disorders, osteoarthritis, cardiovascular disease, and an increased cancer risk, with over 4 million deaths and 150 million DALYs annually on a global scale. Morbidity and disability risks are further increased with sarcopenic obesity, which is characterized by high fat mass and low lean muscle mass. Management can include lifestyle changes, bariatric surgery, and medications. The GLP-1 receptor agonist semaglutide, administered at 2.4 mg once a week, is an effective fat mass reducer with minimal lean muscle loss. Lifestyle interventions can be supplemented by alternatives such as liraglutide and orlistat. The impact of semaglutide on lean mass is an important factor in determining the extent to which the loss is clinically significant or merely an adaptation to weight loss[1].

Semaglutide is a long-acting agonist of glucagon-like peptide-1 receptors that is mainly used in the treatment of obesity and type 2 diabetes. It works by decreasing appetite, delaying gastric emptying, reducing energy intake, and enhancing insulin secretion and glycemic control. Semaglutide can be administered either through weekly injections or taken orally once a day, with no significant difference in efficacy between the two methods. In the clinical trials of the STEP program, semaglutide demonstrated an average weight loss of about 14.9 percent from baseline. The weight loss was primarily attributed to fat mass reduction, with the proportion of lean mass loss ranging between nearly 0 and 40% of the total weight loss. Semaglutide, at a dosage of 2.4 mg per week, decreased body weight by 15.3 kg, comprising 8.36 kg of fat mass and 5.26 kg of lean mass. Fat mass accounted for 61 percent of the total weight loss, while lean mass contributed 39 percent, resulting in an overall increase in the relative percentage of lean body mass[2].

The lean mass loss induced by semaglutide is also raising clinical concerns, although there is evidence that it adapts and does not cause harm. In STEP1, semaglutide produced a mean weight loss of 15.3 kg with a lean mass reduction of 6.92 kg (≈45% of weight lost), demonstrating that substantial lean loss can accompany large pharmacologic weight loss[3]. Importantly, Neeland *et al* conclude that “skeletal muscle changes with GLP1RA treatments appear to be adaptive: reductions in muscle volume seem to be commensurate with what is expected given aging, disease status, and weight loss achieved,” and they report improved muscle quality[4]. Taken together, these studies indicate that semaglutide-related lean loss often reflects expected physiologic remodeling with improved muscle composition, though older or frail patients warrant monitoring and adjunctive strategies (protein and resistance exercise) to preserve function, and individualized risk–benefit assessment is essential in clinical practice.

The disparities in income serve as formidable barriers to access to semaglutide, with persons earning higher incomes and having private health insurance being considerably more likely to take semaglutide. According to the results of the MEPS 2022 data analysis, people who earned more than the median income were 1.61 times more likely to take semaglutide (95% CI: 1.14–2.26), while people with private health insurance were 1.52 times more likely to take semaglutide (95% CI: 1.04–2.22). This points to challenges related to socioeconomic status and insurance coverage. These disparities threaten to increase inequities in diabetes outcomes since lower-income groups experience a disproportionate burden of diabetes but have limited access to care due to affordability, restrictive coverage requirements, and geographic disparities in access. Policy responses need to focus on fair expansions of coverage, targeted subsidies, and reforms in the formulary to provide clinically justified access without income restrictions. At the same time, it is crucial to track real-life adoption, out-of-pocket expenses, and health outcomes among sociodemographic groups to assess interventions and ensure that semaglutide does not exacerbate disparities in diabetes care but instead enhances health equity among underserved and marginalized groups[5].

In conclusion, semaglutide is still an effective obesity treatment that can lead to significant weight loss, mainly by decreasing fat mass without significantly affecting the relative proportion and quality of lean mass. Evidence indicates, at present, that lean mass loss may be more of a physiologic response than a deleterious effect, especially with appropriate protein consumption and resistance training. Nevertheless, the differences in costs and access, as well as insurance restrictions, limit availability, and there is a need to implement equitable policies to ensure semaglutide is accessible to all eligible patients without any disparity.

## Data Availability

Data sharing is not applicable to this article, as no datasets were generated or analyzed during the current study.
